# Qualitative analysis of manual annotations of clinical text with SNOMED CT

**DOI:** 10.1371/journal.pone.0209547

**Published:** 2018-12-27

**Authors:** Jose Antonio Miñarro-Giménez, Catalina Martínez-Costa, Daniel Karlsson, Stefan Schulz, Kirstine Rosenbeck Gøeg

**Affiliations:** 1 Institute for Medical Informatics, Statistics and Documentation, Medical University of Graz, Graz, Austria; 2 Department of Biomedical Engineering, Linköping University, Linköping, Sweden; 3 Department of Health Science and Technology, Aalborg University, Aalborg, Denmark; Hopitaux Universitaires de Geneve, SWITZERLAND

## Abstract

SNOMED CT provides about 300,000 codes with fine-grained concept definitions to support interoperability of health data. Coding clinical texts with medical terminologies it is not a trivial task and is prone to disagreements between coders. We conducted a qualitative analysis to identify sources of disagreements on an annotation experiment which used a subset of SNOMED CT with some restrictions. A corpus of 20 English clinical text fragments from diverse origins and languages was annotated independently by two domain medically trained annotators following a specific annotation guideline. By following this guideline, the annotators had to assign sets of SNOMED CT codes to noun phrases, together with concept and term coverage ratings. Then, the annotations were manually examined against a reference standard to determine sources of disagreements. Five categories were identified. In our results, the most frequent cause of inter-annotator disagreement was related to human issues. In several cases disagreements revealed gaps in the annotation guidelines and lack of training of annotators. The reminder issues can be influenced by some SNOMED CT features.

## Introduction

Standardised terminologies are important resources for interoperability in electronic health records (EHRs) [1,2]. Their use supports hospital reimbursement, audit, research, benchmarking and outcome management [3]. Therefore, quality and accuracy of clinical coding is of utmost importance. A critical assessment of coded data is therefore an important requirement for coding accuracy [4]. Several factors affect the quality of clinical coding. Santos et al. [5] identified some barriers such as poor documentation practices in patient medical records and organisational factors, including unrealistic coding deadlines and inadequate training, among others. According to de Lusignan [6], the attitude of clinicians is a major reason for poor coding. SNOMED CT [7] is a comprehensive multilingual healthcare terminology, which enables machine-processable representation of clinical content in EHRs. SNOMED CT provides terms in different languages, which are linked to language-independent representational units, known as SNOMED CT concepts. Concepts are arranged in subclass hierarchies. Many of them are furthermore described or defined by formal axioms expressible in description logics which qualifies SNOMED CT as a formal ontology [8]. In contrast to other terminologies, only few concepts have text definitions or scope notes attached. The use of SNOMED CT in EHRs is expected to improve communication and semantic retrieval, real-time decision support, cross-border interoperability of health data, and retrospective reporting for research and management [9]. SNOMED CT is particularly relevant for natural language processing (NLP) because (i) it is a reference terminology, which spans many different clinical information (e.g. diseases, procedures, observations …), in contrast to specific application task like ICD-10, which only spans diagnoses; and (ii) large parts of clinical information is only available in narrative form, its use for annotating narratives via natural language processing constitutes an important desideratum and is subject to intensive research [10]. Annotation, in contrast to coding, means the assignment of codes not to directly observed clinical states of affairs but to existing low structured representations thereof like images or texts. Manual annotations of clinical text are important both to produce training resources for NLP systems and for their assessment. To this end, manual annotations need to be accurate. The more human experts disagree about which codes to choose for representing a piece of text, the less interoperability can be expected to result from machine annotations. The goal of the ASSESS CT [11] project was to find empirical evidence for the fitness of SNOMED CT as a potential standard for EU-wide eHealth deployments. For that, among others, we investigated how well it supports the unambiguous annotation of clinical text compared to other terminologies. We obtained as a result of the annotation task a Krippendorffs Alpha [12] value ranging from 0.37 to 0.64 depending on concept matching criteria. This value is lower than 0.8, value required to interpret it as a good consensus between coders [13]. The obtained Alpha coefficient is not unique considering that related coding experiments [14–16] have reported low agreement values as well. In this paper we analyse the coding disagreements between the annotated corpus and a reference standard to obtain a comprehensive list of categories of disagreement. Based on the ASSESS CT experience we provide recommendations to pave the road towards more consistent annotations.

In the Materials and Methods section, we describe the main elements to understand the scope of the ASSESS CT annotation experiment, i.e. the acquisition of the corpus, the recruitment of the annotators and the annotation guideline. Besides, we present the methodology used to carry out the qualitative analysis. Next, the Results section presents the quantitative results of comparing the reference standard with the annotations, and the suggested set of categories for classifying the sources of disagreements that were found. Finally, Discussion section compares our results with related work and Conclusion section provide some annotation recommendations based on the suggested categories of disagreement.

## Materials and methods

In ASSESS CT, the terminology settings for the annotation experiment were subsets of SNOMED CT (January 2016) and the Unified Medical Language System (UMLS) Metathesaurus [17] (without SNOMED CT), covering relevant the semantic groups: Disorders, Objects, Living Beings, Devices, Chemical and Drugs, Procedures, Genes and Molecular Sequences, Concepts and Ideas, and Anatomy. In this work, we only analysed the annotations with the subset of SNOMED CT (see Table 1).

**Table 1:** Number of concepts associated with each semantic group and extracted from SNOMED CT to create the terminology setting.

Semantic group	Content
Disorder	111,424
Objects	312
Living Beings	20,467
Devices	12,822
Chemical and Drugs	5,802
Procedures	55,783
Genes and Molecular Sequences	356
Concepts and Ideas	5,994
Anatomy	28,646

In the following we describe the steps followed for the annotation task: (1) Acquisition of the corpus; (2) Elaboration of the annotation guidelines and (3) Recruitment of annotators. Then, the methodology used for the qualitative analysis is described.

### Acquisition of the corpus

Since ASSESS CT pursued to compare SNOMED CT regarding other terminologies as text annotation tool, the corpus selected should optimise representativeness and diversity of EHR content, in terms of clinical domains, document sections, and document types.

60 medical document snippets (400–600 characters each) in six European languages (Dutch, English, French, Finnish, German and Swedish) including several medical disciplines were selected. They were provided anonymised by project partners and obtained from i2b2 databases. They are now publicly available [18]. All texts had been used previously in other studies and had therefore been manually de-identified. Besides, each snippet was checked for absence of identifying criteria.

Since the corpus was in different languages, a professional translation service [19] was used to produce translations of each snippet into each other language.

### Annotation guideline

Since the guideline was produced in the context of the ASSESS CT project, it was not focused just on SNOMED CT, but it was created with the intent to provide annotators with rules that could be used with any of the terminologies involved. Besides, it had as main driver to obtain the concept coverage and the term coverage of the specified text fragments, which were later used to calculate the disagreement between annotators.

The following terms were defined within the guidelines: (1) Concept: unit of specific meaning in a terminology system (e.g. “Normocytic anemia (disorder)”); Code: alphanumeric identifier for a concept (e.g. 300980002); Token: a single word, a numeric expression or a punctuation sign (e.g. “Modest”); Chunk: Single token or phrase, delineated by the annotator in a way that it corresponds to a clinical concept (e.g. “Modest normocytic anaemia”) and annotation group: (unordered) set of concept codes that jointly represent or approximate the meaning of the clinical concept related to a chunk (e.g. “300980002 |Normocytic anemia (disorder); 255604002 |Mild (qualifier value)|”).

Both, concept and term coverage are provided at the level of a chunk. Inspired by the ISO/TR 12300:2014 [20], concept coverage is used to indicate when a code represents a full, inferred, partial or no coverage of the meaning of a text fragment in a chunk (see Table 2). Term coverage is used to indicate whether the terms of the selected codes from each terminology setting match with slight variations the tokens in the chunks or not (e.g. a positive term coverage with an allowed verb inflection is shown in the text “… the driver suddenly blacked out”. It is annotated with the concept “271594007 | Syncope (disorder)|”, which has the synonym “Blackout”. A negative term coverage is shown in the text “… a coronary heart disease existed and a bypass surgery had been performed…”. The tokens “bypass surgery” were annotated with the code “232717009 | Coronary artery bypass grafting (procedure) |” which does not contain the term “surgery”, but the term “bypass” is posit matched).

**Table 2:** Definition of concept coverage scores for ASSESS CT manual annotation.

Score	Definition
**Full coverage**	When the meaning of a chunk is fully represented by a set of codes, e.g. the term “Heart attack” is fully covered with “22298006 | Myocardial infarction (disorder)|”.
**Inferred coverage**	When the meaning of elliptic or ambiguous chunks of text can be inferred from the context and can be fully represented by a set of codes, e.g. a specific use of the term “hypertension” could mean “Renal arterial hypertension”, so the code “39018007 | Renal arterial hypertension (disorder)|” is justified.
**Partial coverage**	When the meaning of the chunk comes close to the meaning of a set of codes, e.g. “Third rib fracture” is more specific than the code “20274005 | Fracture of one rib (disorder)|”. Yet the meaning is close enough to justify annotation with this code.
**None**	When there is not any set of codes that has a closer meaning to the chunk, e.g. generic codes such as “125605004 | Fracture of bone (disorder)|” for coding “third rib fracture” must not be used as partial coverage.

By following the rational above, the annotation process was described by the following steps:

- Identify the scope of the chunks and assign them a unique number
- Find the most suitable set of codes to represent the meaning of each chunk
- Provide concept coverage scores to each token in a chunk
- Provide the term coverage scores to each token in a chunk.

The scope of the chunks is defined as the tokens that represent a meaningful clinical concept. We mean with "meaningful clinical concept" that the chunk must provide some relevant clinical information, i.e. having a chunk with only “Hyoid bone” or “intact” does not provide any relevant information, but “Hyoid bone is intact” is more informative of the content of the text fragment. Thus, chunks with only body structure or qualifier concepts should not be created, Finally, annotators must use the same scope of the chunk for the different terminology settings.

The general rules for annotating chunks are:

1. First, select the codes that best represent the meaning of a chunk. To this end, the discourse context provided by previous and/or following chunks should be considered.
2. If a chunk requires to be annotated with more than one code, select the lowest number of codes that, together, optimally represent the meaning of the clinical concept.
3. The use of the Averbis Term Platform (ATP) [21], a custom terminology browsing tool is mandatory. Web resources are allowed only to support better understanding of medical terms.
4. If there is a strong doubt about the meaning of a chunk, it should not be annotated.

Given the requirements of the ASSESS CT project, SNOMED CT specific post-coordination syntax was not used, in order to guarantee compatibility with the competing, non-SNOMED scenario. For the same reason, a specific terminology browsing tool was required.

**Fig 1:**
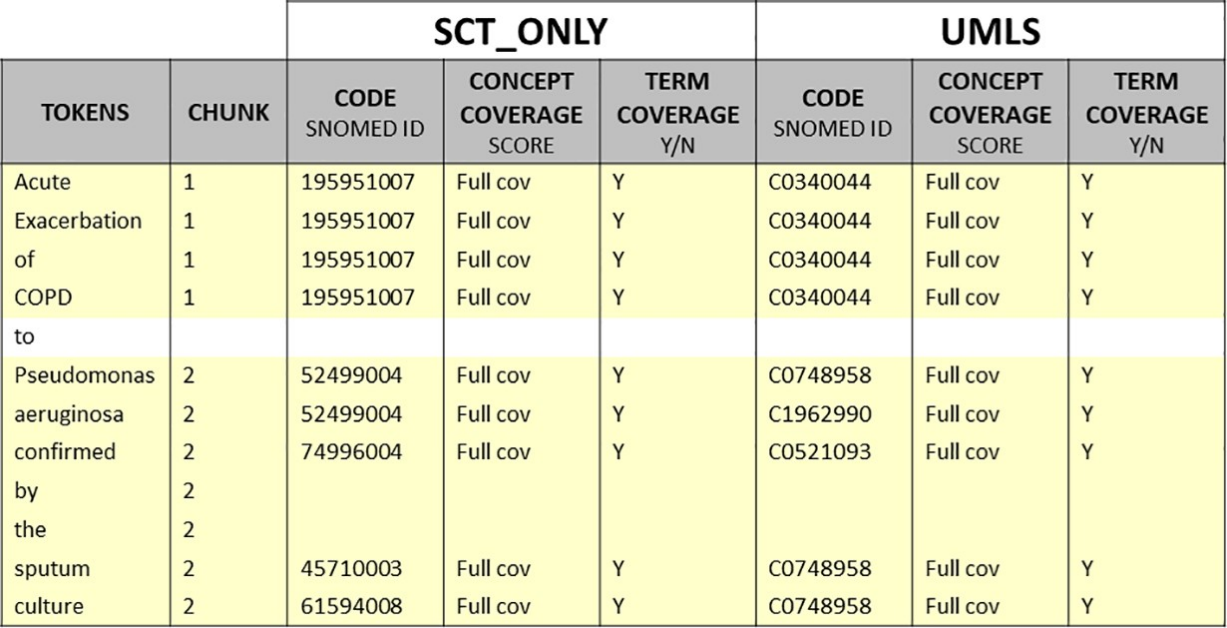
Fragment of an annotation spreadsheet. It shows the header of the document and an example of the annotation of two chunks with SNOMED CT (SCT ONLY) and UMLS terminology settings.

The annotations were carried out using customised spreadsheets. The annotation spreadsheets were designed flexible enough to allow annotators defining chunks and annotation groups but in a standardised way to enable the comparison of the result among the annotators. Fig 1 shows an excerpt of an annotation spreadsheet with two chunks that were annotated with SNOMED CT (SCT ONLY) and UMLS terminology settings. The spreadsheet defines five column types: (1) tokens in the medical document snippet; (2) chunk numbers; (3) selected codes; (4) concept coverage scores; (5) term coverage scores. The empty cells in the spreadsheet indicate that the tokens are out of the scope of the annotation experiment. Consequently, the cells related to both terminology settings must be empty.

### Recruitment of annotators

The accuracy of coding is also related to the medical knowledge of coders. Therefore, physicians who generate clinical data can be the best potential candidates as excellent coders from the aspect of coding performance [22]. In ASSESS CT, annotators were recruited from physicians or medical students during their last academic year and having experience in the use of any of the selected terminologies were not required. For each language, at least two annotators were recruited for the ASSESS CT project. Annotators’ training was guided by the annotation guideline document and a series of webinars that can be obtained from [23] (see S1 File).

### Qualitative analysis

A total of 20 medical text snippets were randomly selected from the corpus at the beginning of the annotation experiment in ASSESS CT to be annotated by every annotator. In this study, we analyse only the annotations of the 20 common medical text of the two English annotators because the versions of SNOMED CT in the other languages are not as complete as the English version in terms of number of concepts or number of synonyms. The annotation results can be consulted in S2 File.

The statistics of the number of concepts associated to the 20 annotated text snippets are shown in Table 3. It quantitatively describes the relevance of each types of concept between both annotators and the reference standard.

**Table 3:** The number of concepts from each semantic group that were used by each annotator and the reference standard for coding the 20 medical text snippets.

Semantic group	Annotator 1	Annotator 2	Reference standard
Disorder	69	98	94
Objects	1	2	2
Living Beings	0	0	0
Devices	2	1	1
Chemicals and Drugs	12	10	10
Procedures	28	31	33
Genes and Molecular Sequences	0	0	0
Concepts and Ideas	68	64	65
Anatomy	67	38	56

In order to be able to assess the coding disagreement between both annotators a reference standard is required. This reference standard was elaborated by two coding experts using the above mentioned 20 text snippets and following the annotation guidelines. The experts considered the annotations already produced and aimed to obtain more precise annotations even if the English annotators agreed. The reference standard is provided in S3 File.

The objective of the qualitative analysis is to investigate the causes of the coding disagreement and to elaborate a comprehensive list of categories which classify them. For that, we compared the codes used to annotate the tokens in every chunk between the reference standard and each of the two English annotators. Due to the possible disagreements in the scope of a chunks between annotators, another delineation was produced during the creation of the reference standard. The reference standard division reconciles the tokens from every annotation group and, therefore, it has a consistent chunk scope.

The resulting categories should consist of the minimum required to cover all disagreement situations, i.e. every disagreement is related to at least one category. Besides, the categories should be related to a specific source of disagreement rather than a situation of disagreement, e.g. the Human issues category groups the disagreement situations related to the lack of medical knowledge and the careless of the annotator to correctly annotate the chunk.

### Limitations

Due to limited time and resources available in ASSESS CT we had to make some concessions. Several limitations arise from these concessions:

- Text fragments or snippets were collected to create a diverse corpus in terms of clinical domain (internal medicine, pathology, etc.), document sections (findings, history, medication, etc.) and document types (autopsy reports, discharge summary, etc.). However, the corpus may not be statistically significant of the medical domain and may limit the impact of the analysis.
- In the construction of the parallel corpus, a translation step was needed to produce the same set of clinical texts in six languages. Despite translation guidelines were given to respect the idiosyncrasy of texts, some language issues were identified, like some acronyms or clinical terms were not correctly translated, and some literal translations were produced instead of finding an equivalent expression in the target language. Few translation errors were also found but they were corrected by the project partners. This could affect some of the disagreement between annotators.
- The annotation experiments were focused on a subset of clinical concepts, mainly, to reduce the amount of coding time and annotators effort. Besides, SNOMED CT concept model and post-coordination method were omitted from annotation guidelines. However, the qualitative analysis is based on the selected clinical concepts and not the whole content of SNOMED CT. So, there could be missing categories or source of disagreements that were not found in this experiment.
- The list of proposed categories aims at summarizing the source of disagreements. Since annotators did not participate in the qualitative analysis, the accurate reasons of disagreement could not be tagged. Thus, this limits the scope of the categories when trying to condense the possible reasons in a consistent way.

## Results

Table 4 shows the quantitative analysis of the agreement of each annotator and the reference standard. More specifically, it shows how many chunks in the reference standard agree with: (i) annotations of the two English annotators; (ii) annotations of the first annotator but not with the second annotator; (iii) annotations of the second annotator but not with the first annotator and (iv) neither first nor second annotator. We consider two chunks agree when they contain the same concepts codes.

**Table 4:** Quantitative analysis of the agreements at chunk level between the reference standard with the two English annotators.

Cases	Agreements
Reference standard agrees with both annotators	50
Reference standard agrees only with the first annotator	39
Reference standard agrees only with the second annotator	50
Reference standard does not agree with any annotator	92

The corpus was split into 231 chunks. We can observe in Table 4 that the reference standard has 92 chunks which none of the annotators agreed with, including 17 cases where the annotators agreed with each other. The code coincidence between annotators with the reference standard was astonishingly low: 21.6% of the chunks the annotation groups include the exact same SNOMED CT codes among the two annotators and the reference standard.

Next, we describe in detail the five categories established to represent the main sources of disagreement between the annotators from more to less frequent. Table 5 shows them together with their frequency. Note that more than one source of disagreement could affect the same chunk, so in some cases a chunk was associated with more than one disagreement category.

**Table 5:** Typology of disagreements and their frequency.

Categories of disagreement	Frequency
Human issues	156
Annotation guidelines issues	38
Ontology issues	22
Interface term issues	22
Language issues	9

### Disagreement categories

**Human issues.** This category encompasses all type of disagreements due to human issues. The three identified causes of such disagreements may be related to the lack of domain knowledge, inability of annotators to decipher medical short forms or just to carelessness because of time pressure and/or lack of intrinsic or extrinsic motivation. Although, annotators were recruited from physicians or medical students during their last academic year, the text came from diverse medical disciplines in which the annotators may not have enough background experience. The different causes of human issues are exemplified below. Even experienced domain expert annotators may fail deciphering certain idiosyncratic short forms such as in “MVT qd” which corresponds, according to the medical text, to the concepts “Thiotepa (substance)”, “Mitoxantrone (substance)”, “Etoposide (substance)” and “daily (qualifier value)”, but it was annotated with “Multivitamin agent (substance)” and “daily (qualifier value)”. The use of text snippets made this situation worse as the lack of context was frequent. In addition, the usability of the retrieval tool played an important role just as the richness of the terminology in synonyms or entry terms. An example how the term retrieval interface influenced the result could be shown with the fragment” …treatment for intractable pain …”, annotated with “Chronic intractable pain (finding)” in the reference standard. This code is not found using the ATP tool if the wild card character “* intractable pain” is not added. Annotators were trained to correctly use the features of the ATP browsing tool but sometimes they did not use it.

Most of disagreements were simply due to carelessness, such as typos or copy/paste errors. Usually, missing guideline compliance occurred because annotators did not follow our annotation guideline recommendation of optimising the annotation to yield the lowest number of codes per chunk, giving preference to pre-coordinated, fully defined concepts. One example is the text “The qualitative drug screening of the urine …”, annotated by “Qualitative (qualifier value)”, “Drug screening test (procedure)” and “Urine specimen (specimen)”, although the meaning of the latter two concepts corresponds to “Urine drug screening (procedure)”.

#### Annotation guideline issues

Well-crafted annotation guidelines are needed for good annotation agreement. This category covers only the causes of disagreement that can be linked to underspecifications of the guideline. For example, our guideline gives preference of active ingredient code(s) over corresponding product code, wherever possible (such as “Aspirin (substance)” instead of “Aspirin (product)”). A similar recommendation aims at reducing disagreement by clear preference criteria for body structure concepts of the type “Structure of X” over “Entire X”, e.g. “Hip region structure (body structure)” over “Entire hip region (body structure)”. In other cases, we found out that our guideline missed specific recommendations to deal with some identified cases of annotation variability. For example, we only detected after the experiment that no selection criteria were specified between “Clinical finding” and an “Observable entity” in cases that lacked a “Qualifier value” in the text, such as in the example: “Give the patient an NaCl infusion guided by the blood pressure”. One annotator annotated “blood pressure” with “Blood pressure finding (finding)” whereas the other one gave preference to “Systemic blood pressure (observable entity)”. Further analysis of the taxonomic structure of the clinical findings hierarchy suggested that “Blood pressure finding (finding)” here plays the role of a merely navigational concept, i.e. an organising node under which really meaningful concepts like “Normal systolic blood pressure (finding)” are grouped. “Blood pressure (observable entity)” would be more adequate, although “Blood pressure taking (procedure)” could also be discussed. Clear guidance is therefore needed. As similar case is the competition between “C-reactive protein measurement (procedure)” and “C reactive protein (substance)”.

#### Ontology issues

This category of disagreements is related to the impact of the unique characteristics of SNOMED CT in contrast to other medical terminologies. The most common reason of disagreements related to ontology issues is the polysemy of clinical terms of the related concepts which belong to different hierarchies in SNOMED CT (only distinguished by the hierarchy tag in the fully specified name). The meaning of concepts in each hierarchy is well defined in SNOMED CT, but it is complicated to identify in the text which is the best concept candidate. Without a clear understanding of the text, a comprehensive list of recommendations and a deep knowledge of the terminology, annotators tend to disagree about which code to select. This is very typical for the hierarchies “Disorder” vs. “Morphological abnormality” or “Clinical finding” vs. “Observable entity” vs. “Procedure”. For instance, the text “Malignant lymphoma” could be annotated with either the concept “Malignant lymphoma—category (morphologic abnormality)” or the concept “Malignant lymphoma (disorder)”. This phenomenon, known as inherent polysemy of so-called “dot-objects” [24], arises only when there are ontological dependency relations involved, like in this example where “Malignant lymphoma (disorder)” is related to “Malignant lymphoma—category (morphologic abnormality)” via the (existentially qualified) relation “Associated morphology”. Thus, the dot-object “Malignant lymphoma” is associated to the dot-type morphology disorder. Since, in our experiments the ontological relations of SNOMED CT were excluded for setting a fair evaluation scenario the agreement between annotators was negatively affected by dot-type codes, i.e. Alfa coefficient was not calculated by expanding the codes related to dot-types.

**Fig 2:**
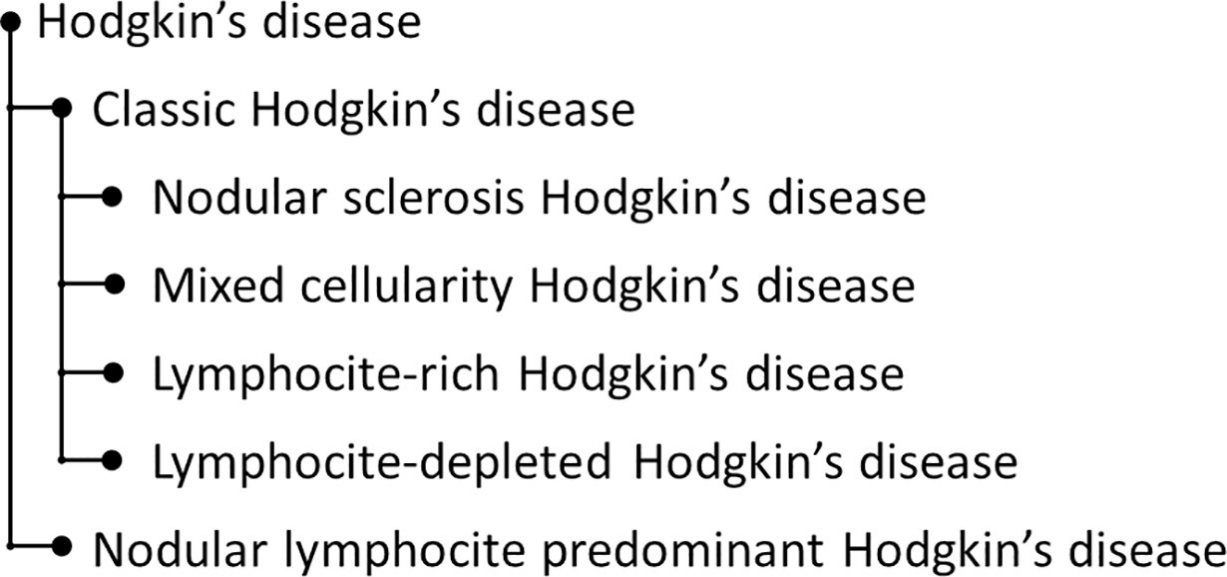
Hodgkin's disease hierarchy.

The existence of concepts, Navigational concepts, that exist only to support the process of location a concept following routes through SNOMED CT as well as the annotation with high-level concepts from SNOMED CT is another source of disagreements. These codes represent generic nodes in SNOMED CT hierarchies that bring together codes that represent a specific clinical concept. For example, “voice normal” is annotated in the reference standard using the concepts “Normal (qualifier value)” and “Voice production finding (finding)” whereas an annotator assigned the parent concept “Voice finding (finding)”. Related with the previous issue is a phenomenon we call here pseudo-polysemy. As an example, the text “Former smoker” was correctly annotated with “Ex-smoker (finding)” by one annotator whereas the other one used the combination of “Smoker (finding)” with “In the past (qualifier value)”. This suggests a polysemy of the word “former” with “ex-” and “in the past”. In fact, what happens here is that there are two annotations that have mostly the same meaning at face value; whereas neither concept exhibits any text definition, so that their exact meaning remains unclear. The temporal scope of “in the past” may extend until the present is also merely speculative. Incomplete concept definitions are still pervasive in SNOMED CT [25], especially when qualifier values are involved. This is a source of coding disagreement because there is not any logical co-ordination mechanism in SNOMED CT to link them. For example: the above example: “Ex-Smoker (finding)” is not defined by any temporal attribute. Another example is “Diabetes monitoring”, annotated with “Diabetes mellitus (disorder)” and “Monitoring action (qualifier value)”, as well as with “Diabetic monitoring (regime/therapy)”. Again, the latter concept is primitive and unrelated to “Diabetes mellitus (finding)”. Analogously, “Skeletal muscle normal (finding)” is unrelated to “Normal (qualifier value)”. The underspecifications of qualifiers in SNOMED CT is also a source of disagreements. The “Qualifier value” hierarchy, whose ontological heterogeneity is a well-known fact, encompasses a large number of qualifiers classified according to different, not always transparent, criteria. Depending on what is measured “Slight (qualifier value)” represents a magnitude whereas “Mild (qualifier value)” represents a severity degree. This often clashes with the use of words like in “The examination revealed slight bleeding in the area of the mitral valve”, interpreted as (quantifiable) amount of bleeding by one annotator and as (qualitative) bleeding severity by the other one. Although, SNOMED CT concept model restrict how to use severity degrees and magnitudes, annotators must interpret which is the most appropriate code. It often becomes more complicated when considering both fully specified name and synonyms of the concept. Finally, some disagreements pointed at missing content in SNOMED CT. For example, we could not annotate accurately the term “Classic Hodgkin lymphoma” in “Histology confirmed the diagnosis of a classic Hodgkin lymphoma from a cervical lymph node” because this concept was missing in the SNOMED CT taxonomy. Thus, the annotators had the dilemma of choosing the parent concept or guessing the most likely one of the child nodes. Fig 2 shows a more precise hierarchy structure of Hodgkin’s disease according to [26] whereas SNOMED CT taxonomy provides 18 child nodes of Hodgkin’s disease which are further expanded.

#### Interface term issues

Interface terms provide close-to-human expressions, including synonyms and abbreviations [27]. In contrast to SNOMED CT fully specified names and preferred terms, the English and Spanish releases represent clinical jargon as synonyms, which can be used as interface terms. Our analysis showed that different SNOMED CT concepts had similar interface terms, which complicated the choice, e.g. “Worried (finding)” and “Anxiety (finding)” are different concepts with the first one having “Anxious cognitions” as interface term. Another example is that “Pain (finding)” has “Pain observation” as an interface term. Consequently, it sounds very similar to “Pain observable (observable entity)” which is an “Observable entity”. A similar confusion appeared with the interface term “Blood extravasation” for “Hemorrhage (morphologic abnormality)”. Again, Hemorrhage belongs to those concepts that are primitive although they could and should be fully defined, e.g. in the example with “Blood (substance)” and “Extravasation (morphologic abnormality)”.

#### Language issues

Linguistic features like ellipsis, anaphora and paraphrase make it difficult to parse clinical texts. However, this characterises the sublanguage of clinical narratives and causes interpretation problems. For example, “Aneurysm of the internal iliac was treated inside the artery by embolization” contains an ellipsis. Annotators should–according to our guideline–understand the text and choose “Structure of internal iliac artery (body structure)” instead of “Arterial structure (body structure)” for the word “artery”. In “Tongue deviates slightly to the left but full strength” the word “muscle” was omitted and should be interpreted and annotated with “Normal muscle power” and “Structure of muscle of tongue (body structure)”. Other linguistics features like coordination also affect the annotation agreement rate. For example, “wounds on the left eyelid and chin” whose annotation in the reference standard includes the concepts “Open wound of eyelid(disorder)” and “Open wound of chin (disorder)” instead of “Open wound of eyelid (disorder)” and “Chin structure (body structure)”. Negation also often requires interpretation efforts to get the best representation such as in “Absence of chest clinical and radiological impact” best represented, according to the authors, by “On examination—chest examination normal (finding)” and “Standard chest X-ray normal (finding)” instead of using “Chest injury (disorder)” and “Standard chest X-ray normal (finding)”.

## Discussion and related work

Continuous assessment of clinical coding is required to find ways to improve its accuracy [28]. In Andrews et al. [14], a study comparing the coding consistency among three professional SNOMED CT coding companies showed no significant agreement among the experts with 33% of agreement on the same concepts. This result is similar to the 21.6% of chunks with complete agreement of their codes among the two annotators and the reference standard.]. Andrews et al. defined preferred hierarchies of SNOMED CT to avoid the ambiguity of dot-objects when selecting the codes (see section “Ontology issues”). It also suggested a possible cause of disagreement due to coded data not being syntactically and semantically clear, which can be related to our suggested category “Language issues”. Other studies also suggest that the agreement may be relatively low among physicians and coding professionals [29]. These results coincide with the Alpha coefficient that we obtained in ASSESS CT coding experiment of medical text with SNOMED CT by two coders. Chiang et al. [30] describes a study to measure the SNOMED CT coding agreement among three physicians, which aimed at measuring the influence of a terminology browser used for coding, showing a full inter-coder agreement of 44% and 53% according to browser selected. It concluded that the SNOMED CT coding could be significantly affected by the specific browser used for modelling. Our results showed similar situation with the ATP browser that contributed in some cases of disagreement, which might have been avoided by a browser that does not require wildcard characters and strict word order, such as the SNOMED CT Web browser. It evidences the need of the qualitative study for investigate the typical categories of disagreements in coding experiments. In Hwang et al. [31] the inter-coder agreement was measured for ophthalmology cases parsed into discrete concepts, using five terminologies, including SNOMED CT, with three physicians serving as annotators. For SNOMED CT, there was an agreement level of 44% among the three annotators. The authors interpret the poor agreement result by proposing that a combination of physician training, terminology refinement, supported by more sophisticated terminology browsers may be necessary. Although, some studies have reported the lack of actual improvement of intercoder reliability due to coder training [32], the shortcomings and underspecifications of the coding guidelines have an influence on the resulting agreement in our experiment. In this study, we investigated the causes of each disagreement and classify into a disagreement category. However, we analysed the disagreements at chunk level of the two annotators against the reference standard. Thus, several categories could be assigned to each chunk. Consequently, the frequency of disagreement categories is higher than the number of chunks analysed. For example, the chunk “Cranial nerve IX and X: voice normal, palate elevates symmetrically” was classified into Human issues because one annotator did not find any code for “voice normal” but “Voice production finding (finding)” and “Normal (qualifier value)” where in ATP browser. Besides, the chunk was also assigned to Annotation guideline issues because the guidelines did not provide any recommendation between the use of the procedure over the finding concepts, therefore, an annotator used the concept “Ninth cranial nerve finding (finding)” instead of “Examination of cranial nerve” and “Cranial nerve IX” like in the reference standard. The set of disagreements grouped in human issues category makes it the least actionable category. However, we could not divide it into more fine-grained categories without increasing the uncertainty of the causes of disagreement, because no feedback was provided by the annotators during the analysis.

Other studies pursue the automatic annotation of medical texts with SNOMED CT using machine learning methods [33] or natural language processing methods [34]. These approaches may solve many of the issues described in the “Disagreement categories” section, such as disagreements and errors due to carelessness of annotators or ambiguities due to similar interface terms. Other issues from the categories “Annotation guideline issues”, “Ontology issues” and “Language issues” must be considered during the development of the automatic methodologies. Further studies are needed to discover the categories that complicate an automatic approach. We believe that issues related to the correct interpretation of the complex medical language may hinder the selection of the most suitable code or SNOMED CT expression.

## Conclusions and recommendations

The usefulness of terminological and/or ontological standards for achieving semantic interoperability critically depends on how consistently they are used, both in human and machine annotation scenarios. This can be measured by the agreement between annotators. Only a sufficiently high inter-annotator agreement leads to accepted content retrieval rates in diverse application scenarios. In addition, a high agreement between human annotators is required for annotated training resources from which automated annotators are trained. In an annotation exercise, done in the context of testing the fitness for purpose of SNOMED CT, the analysis of annotations of clinical text samples, done by medically trained annotators, highlighted typical categories of annotation errors and disagreements. The most frequent error category was due to human issues like trivial mistakes, unsuccessful term retrieval, limits of domain knowledge and non-compliance with the guideline. The second most frequent category of disagreement was related to guideline underspecifications. Although some of these problems highlighted known SNOMED CT issues they should have been anticipated as guideline recommendations. The third category contains errors due to ontology issues in SNOMED CT, such as inherent polysemy due to dot-types, pseudo-polysemy, unspecified SNOMED CT concepts, especially in the qualifier value hierarchy. The next category addresses interface term issues, related to problems associated with the synonyms in SNOMED CT, especially relevant for term retrieval. Our analysis of the disagreements in this category highlights the problems of annotators in selecting the right code based on the interface terms provided. The last and smallest category of disagreements is related to specific language issues like ellipsis, anaphora and paraphrase. Although full annotator agreement annotation is an unrealistic desideratum, our error analysis may pave the road towards more consistent annotations. We recommend the following:

- The quality of SNOMED CT requires improvement in multiple aspects. It includes the provision of formal axioms wherever possible, the conversion of primitive concepts into fully defined ones. Where this is not possible, the meaning and the intended use of concept needs to be specified by scope notes or text definition. In our understanding, it is prohibitive for a standard like SNOMED CT to tolerate ill-defined concepts whose interpretation and delineation is incumbent on the user. There should also be a mechanism for dealing with polysemy due to dot-types like morphology●disorder or finding●observable. Currently, SNOMED CT provides the mechanism Right Identities, also called property chains, to address this issue between product●substance using the Description Logic Transform to OWL script [35] from SNOMED CT. Given that a certain degree of disagreement is unavoidable, terminologies based on a logical model like SNOMED CT should at least be able to identify diverging annotations that are semantically equivalent. As we have demonstrated primitive concept, which could be fully defined impair the recognition of equivalent expressions. In our data, there was no one example where disagreement could be resolved by logical reasoning.
- Annotation guidelines should offer more complete set of recommendations that tackle the most frequent potential annotation disagreements. In our experiments, the lack of recommendations of annotation preferences which guide the annotators among hierarchies, such as give preference for identifying diagnoses or procedures, produced several disagreements that could be avoided.
- Annotations require highly qualified experts, but the annotation process may be considered boring, with the risk of annotators getting absent-minded, which leads to a decrease in annotation quality. Apart from intensive training, intrinsic and extrinsic motivation of the annotators should be optimised.
- Tool support is essential. User-friendly, powerful term retrieval and annotation tools can contribute to reduce disagreements due to human and language issues. De Lusignan [6] highlights that those large and sophisticated terminologies such as SNOMED CT offer a large choice of concepts but also potential problems with searching. But tool support is not limited to offer better retrieval comfort. So, an advanced annotation toolset could include a real-time checking of guideline constraints in order to early detect certain (probably not all) guideline violations. Tooling should also support the annotation workflow. E.g., by aligning pairwise annotation processes, disagreement between pairs of reviewers can be readily detected. Finally, incentives (credits for good annotations) or gamification of the annotation process could increase attentiveness and motivation.

## Supporting information

S1 File Annotation guidelines. This file contains the annotation guidelines followed by the annotators.
(PDF)

S2 File Annotated texts. This spreadsheet contains the results of the annotation experiment in a single table. The columns indicate: (1) the language of the annotated text; (2) the identifier of the annotator; (3) the identifier of the text snippet; (4) the sentence identifier in the text; (5) the annotated text token; (6) the chunk number; (7–9) the list of codes, concept coverage score and term coverage for SCT_ONLY scenario; (10–12) the list of codes, concept coverage score and term coverage for UMLS_EXT scenario; (13–15) the list of codes, concept coverage score and term coverage for LOCAL scenario.
(XLSX)

S3 File Qualitative analysis results. This file contains the results of the qualitative analysis of the English annotations with reference standard and their associated categories.
(XLSX)
