# Supplementary material for: Qualitative analysis of manual annotations of clinical text with SNOMED CT
Source: PLoS One. 2018 Dec 27;13(12):e0209547. doi: 10.1371/journal.pone.0209547 (PMC6307753; doi:10.1371/journal.pone.0209547)
Supplement: S1 File — This file contains the annotation guidelines followed by the annotators. (PDF) [file pone.0209547.s001.pdf]

Annotation guideline for ASSESS-CT

Version: September, 1st, 2015

1. Introduction

This annotation guideline is created for use in ASSESS CT. One of the goals of this project is to represent part of the meaning of medical narratives by terminology codes, obeying certain coding restrictions. The guidelines have been optimised to compare different terminology settings in a limited corpus. Consequently, they are not optimized for real coding scenarios.

ASSESS CT has prepared clinical text snippets in different languages, which are annotated within at most three terminology scenarios, named SCT_ONLY, UMLS_EXT, LOCAL.

2. Definitions

Regarding the naming in the annotation guidelines the following notions will be introduced:

- **Concept**: entity of independent domain specific meaning in a terminology system.
- **Code**: alphanumeric identifier for a concept.
- **Token**: a single word, a numeric expression or a punctuation signs. A token is obtained by splitting the words in a text using white spaces and punctuation signs.
- **Chunk**: A chunk is a text fragment consisting of one or more tokens. A chunk is delineated in a way that it corresponds to a clinical concept. The delineation of a chunk may depend on individual judgement.
- **Annotation group**: (unordered) set of concept codes that jointly represent or approximate the meaning of the clinical concept related to a chunk.

3. Resources

The annotation task requires two resources:

- Excel spreadsheets where each tab corresponds to one text sample to be annotated for a single language.
- A Web-based terminology server that supports the retrieval of codes for the three terminology scenarios and six languages, Averbis term browser (http://apps.averbis.de/atp/).

4. Workflow

The annotators will be given tailored spreadsheets where each tab corresponds to one sample to be annotated for a single language. A spreadsheet is divided into the columns (i) Tokens; (ii) Chunks; (iii) SCT ONLY; (iv) UMLS EXT; and (v) LOCAL (Figure).

Once the annotators receive their spreadsheets, the token column is already filled. Furthermore, each spreadsheet has a unique identifier for each annotator.

As a first step, the annotator identifies the chunks. Relevant chunks are those that contain medical terms relevant to the scope of the terminologies used for annotation (see section 6).

The columns SCT ONLY, UMLS EXT, and LOCAL correspond to three different annotation scenarios. For each scenario a different terminology setting is activated in the Term browser. One or two scenarios may be disabled, according to language-specific settings.

Each annotation scenario requires three columns to be filled: (i) the list of terminology codes; (ii) concept coverage score (see Table 1); (iii) term coverage score (yes / no). For each chunk, appropriate codes are entered.

Three options exist for filling out the spread sheet:

1. If a token is not part of a relevant chunk then the corresponding field remains empty (see line 1 in Figure),
2. If the meaning of the chunk is fully represented by a code, then this specific code is entered (see lines 13 - 16 in Figure)
3. When there is an overlap between two concepts within the same token (see example in Figure) or the token is related to several clinical concepts, then all of the relevant codes are entered (see example in Figure).

	TOKENS	CHUNK	SCT ONLY			UMLS EXT			LOCAL		
			**CODE** SNOMED ID	**CONCEPT COVERAGE**	**TERM COVERAGE**	**CODE** UMLS CUI	**CONCEPT COVERAGE**	**TERM COVERAGE**	**CODE** Local Term	**CONCEPT COVERAGE**	**TERM COVERAGE**
1	A										
2	40-year-old										
3	female										
4	with										
5	history										
6	of										
7	non-ST-elevation	1	401314000	Partial cov	no	C1561921	Full cov	yes		No cov	no
8	myocardial	1	401314000	Partial cov	yes	C1561921	Full cov	yes		No cov	no
9	infarction	1	401314000	Partial cov	yes	C1561921	Full cov	yes		No cov	no
10	in										
11	2016-09-30										
12	with										
13	stent	2	216621000119100	Full cov	no	C3836455	Full cov	no		No cov	no
14	to	2	216621000119100	Full cov	no	C3836455	Full cov	no		No cov	no
15	the	2	216621000119100	Full cov	no	C3836455	Full cov	no		No cov	no
16	LAD	2	216621000119100	Full cov	no	C3836455	Full cov	no		No cov	no
17	and										
18	50%										
19	to										
20	the										
21	mid	3	91748002	Full cov	no	C1321506	Partial cov	no	X74eE	Partial cov	no
22	LAD	3	91748002	Full cov	no	C1321506	Partial cov	no	X74eE	Partial cov	no
23	,										
24	had										
25	instent	4	251030009	Partial cov	no	C1868718	Inferred cov	no		No cov	no
26	restenosis	4	43026009	Full cov	yes	C1868718	Inferred cov	no		No cov	no
27	in										
28	2017-04-02										
29	and										
30	then										
31	underwent										
32	brachytherapy	5	399315003	Full cov	yes	C0006098	Full cov	yes	8J0..	Full cov	yes
33	to	5			no			no			no
34	the	5			no			no			no
35	RCA	5	362037006	Full cov	yes	C1261316	Full cov	yes	X74eO	Full cov	yes
36	,										
37	who										
38	presented										
39	to										
40	Baldpate										
41	Hospital										
42	with										
43	several										
44	weeks										
45	of										
46	chest	6	139228007	Full cov	yes	C0008031	Full cov	yes	182..	Full cov	yes
47	pain	6	139228007	Full cov	yes	C0008031	Full cov	yes	182..	Full cov	yes
48	similar										
49	to										
50	her										
51	anginal	7		No cov	no	C0741034	Full cov	yes		No cov	no
52	equivalent	7		No cov	no	C0741034	Full cov	yes		No cov	no
53	and										
54	MI	8	22298006	Full cov	yes	C0027051	Full cov	yes	X200E	Full cov	yes
55	in										
56	the										
57	past										
58	.										
59	It										
60	started										
61	at	10	263678003	Full cov	yes	C0443144	Full cov	yes	XC062	Full cov	yes
62	rest	10	263678003	Full cov	yes	C0443144	Full cov	yes	XC062	Full cov	yes
63	.										
64	No										
65	relief	11	182970005	Inferred cov	no	C0451615	Inferred cov	no	8BAA.	Inferred cov	no
66	with	11			no			no			no
67	nitroglycerin	11	387404004	Full cov	yes	C0017887	Full cov	yes	bl1..	Full cov	yes
68	x3										
69	.										

Figure 1. Example of annotation spreadsheet.

It is recommended that the relevant chunks are highlighted in the spread sheet using a yellow background colour such as in Figure 2. Mandatorily, each chunk that contains annotations needs to be identified by a distinct number.

5. Annotation phase

For each token, the following information is entered by the user into the annotation spreadsheet.

- **The corresponding code(s)**. These codes are obtained by performing a search in the Term browser, limited to the corresponding language and coding scenario. If there is no immediate hit, searches by synonyms and substrings should be performed. In the rare case that the meaning of a single token corresponds to two or more codes (which may occasionally be the case with lengthy single-word compounds in German, Dutch or Swedish), these codes are all entered in the same cell, separated by semicolons.
- **Concept coverage**. Scores are assigned to each token in a chunk. If a token is out of scope of the annotation, the concept coverage score could be empty. If a code covers more than one token in a chunk, then it is indicated in each token (including articles, prepositions) their corresponding coverage score. If the meaning of a token needs to be represented by more than one code, the score to be used corresponds to the lowest coverage score of those codes.

Table 1 shows the allowed concept coverage values:

Table 1 . Values of concept coverage score

Rating	Meaning
1- **Full cov**	Full coverage: The meaning of the text fragment fully represents the concept. E.g. The term "high blood pressure" is fully covered by "Hypertensive disorder" using the SNOMED CT code 38341003.
2- **Inferred cov**	Inferred coverage: Although the text fragment is elliptic or ambiguous, its meaning can be inferred from the context and can be fully represented by a concept. E.g. a specific use of the term "hypertension" could mean "Renal arterial hypertension", so that the annotation with the SNOMED CT code 39018007 is justified.
3- **Partial cov**	Partial coverage: The meaning of the text fragment comes **close** to the meaning of the concept. E.g. "Third rib fracture" is more specific than what can be found in the terminology, namely "Fracture of one rib" with the SNOMED CT code 20274005). Yet the meaning is close enough to justify annotation with this code.
4- **No cov**	No coverage: There is not any concept that comes close to the meaning of the text fragment. Too unspecific concepts such as "fracture of bone" for "third rib fracture" must not be used for "partical cov". Here, "no cov" is the correct decision.
5- **Out of scope / EMPTY**	The meaning of the text fragment is not covered by any of the semantic groups selected for this study.

- **Term coverage.** It is annotated with Yes/No values for each token in an annotated chunk. If the token occurs in the term of the terminology literally or with minor variants (inflection, word order, typing error), then it is considered a full match and is annotated with YES. Any other situation is a NO. There will be no distinctions between all synonyms, or entry terms of concepts. There could be a partial conceptual coverage but a full term match. Thus, this is a typical situation when there is a high level of ambiguity in a terminology.

6. General guidelines

The annotation of medical text is restricted to Findings, Procedures, Substances, Results of clinical and lab measurements, including related qualifiers, organisms, medical devices and body structures.

The goal of the first task is the delineation of chunks, which must the same for all annotation setting (SCT ONLY, UMLS EXT and LOCAL). The goal of the second task is finding the best codes in Averbis Term browser and assigning the corresponding concept coverage score. The goal of the final task is to add the term coverage score.

A. <u>General rules for chunking process:</u>

- Each chunk should represent a meaningful clinical concept in the text.
- The clinical concept has to be related to, at least, one of the selected semantic groups.
- The chunks must have associated a distinct number and it is also recommended to shade the cells with a yellow background.

B. <u>General rules for annotation:</u>

- The annotators must select the concept codes that better cover the meaning of the clinical concept in a chunk. The full coverage and inferred coverage scores has the same coverage level.
- If a chunk needs to be annotated with more than one code. The annotator must select the fewest number of codes that, together, better covers the meaning of the clinical concept. Only partial overlap of the tokens covered by the codes is allowed.
- The annotators must not use other browsers than AVERBIS term browser and web Wikis to find the correct codes.
- If there is a doubt about the meaning of tokens in a chunk they must not be annotated. These tokens have to be kept empty.

For example the chunk “Complicated fracture of third rib” can be annotated with the following codes:

1. |255302009| Complicated.
2. |706922007| Complicated fracture of bone.
3. |125605004| Fracture of bone. If annotators are more literal this code could be full coverage. However, other could see the next code as more appropriate and also use the score "inferred coverage".
4. |20274005| Fracture of one rib.
5. |25888004| Bone structure of third rib (body structure).

As a consequence, the possible annotation sets with full coverage within a single chunk could be:

i. [1, 3, 5]
ii. [1, 4, 5]
iii. [2, 4, 5]
iv. [2, 5]. This would be the best annotation group because fully coverage the content of the chunk and also has fewer number of codes.

The annotators should not annotate the following content:

- Proper names, persons, professional roles, social groups, geographic entities, institutions, non-medical devices, non-medical events.

- Context such as diagnostic certainty, plans, risks, clinical history, family history. For instance, in the phrase “high risk for lung cancer” only “lung cancer” is annotated, as well as in “father died from lung cancer”, or “suspected lung cancer”.
- Temporal information. E.g., in the phrase “lung cancer, first diagnosed in Oct 2014” only “lung cancer” is annotated. In “old MI”, only “MI” is annotated (even if there is a concept for “old MI”)
    a. The only case where temporal information is annotated is where it is part of a drug prescription such as “1-1-1” or “t.i.d.”
- Residuals, e.g. "Arterial hypertension NEC", "Tuberculosis, unspecified", "other complications of unspecified head injuries"
- Numerals, e.g. "eight".

The following preference conditions should always be considered:
- Anatomy concepts that contain the word “Structure” should be given preference about those that contain the term “entire” in their preferred terms.
- Finding / disorder concept should be given preference over corresponding morphology concepts.
- For all lab values, preference should be given to those concepts that include the term “measurement”, such as “measurement of potassium in serum”.

C. General rules for term coverage:
- The tokens annotated in a relevant chunk have to be annotated with “yes” or “no” term coverage.
- The term coverage is not case sensitive and does not need to match punctuation marks.
- There is a term coverage “yes” if the token occurs in the term of the terminology literally or with minor variants (inflection, word formation, word order, typing error).
- Acronyms that are in the terminology part of a term, such as “MI - Myocardial infarction”, be used.
- If more than one token is covered by one term in the terminology, it is possible that some tokens are annotated with “yes” and other with “no”. (See Figure 8)

Examples of annotation groups

Figure shows the token “Oesophagitis” that belongs to the chunk #1 and is annotated by the SNOMED CT code 8765009. The code fully covers the meaning of the token. There is also full term term coverage, because the concept is associated with the term “Oesophagitis” in the term browser. This is the simplest annotation case.

TOKENS	CHUNK	CODE SNOMED ID	CONCEPT COVERAGE	TERM COVERAGE
Oesophagitis	1	16761005	Full cov	yes

Figure 2. Single code annotation

In the Figure example a chunk has two tokens “Ulcerated” and “esophagitis”. The code corresponds to “Ulcerative esophagitis” concept and fully covers the meaning of the chunk.
Again, the rating is “full concept coverage” and “term coverage = yes”. Note that it is required to fill

all fields in the chunk; in this case with the same values. The interpretation is that the meaning of the chunk is given by one single concept.

TOKENS	CHUNK	CODE SNOMED ID	CONCEPT COVERAGE	TERM COVERAGE
Ulcerated	1	439955006	Full cov	yes
esophagitis	1	439955006	Full cov	yes

Figure 3. Annotation with a single concept, denoted by two tokens.

Figure shows the annotation of "haemoglobin" and "decrease", two tokens constituting a single chunk. The meaning of either token is fully covered by the codes 38082009 and 260370003, respectively. In both cases the tokens correspond to terms found in the term browser as belonging to the respective concept. The meaning of the chunk is represented by the group {38082009, 260370003}. Groups aggregate concepts that belong to the same chunk. They have the properties of a mathematical set: there is no order and duplicates are ignored.

TOKENS	CHUNK	CODE SNOMED ID	CONCEPT COVERAGE	TERM COVERAGE
haemoglobin	1	38082009	Full cov	yes
decrease	1	260370003	Full cov	yes

Figure 4. Annotation with two concepts.

In Figure a partial overlap between concepts is demonstrated. Here, "esophagitis" occurs in both terms "Ulcerative esophagitis" and "Esophagitis grade II". A term "ulcerative esophagitis grade II" does not exist. As in Fig.3 the meaning of the chunk has to be assembled by existing concepts. In this case, however, this leads to an overlap, because "esophagitis" occurs in both concepts. This explains that two codes are entered into the second field. The meaning of the chunk is represented by the group {439955006, 413226009}. Term coverage is given in both cases. In case the first term was "erosive esophagitis", term coverage is not given. In the second line (occupied by two concepts), the joint coverage would be "no".

TOKENS	CHUNK	CODE SNOMED ID	CONCEPT COVERAGE	TERM COVERAGE
Ulcerated	1	439955006	Full cov	yes
esophagitis	1	439955006; 413226009	Full cov	yes
grade	1	413226009	Full cov	yes
II	1	413226009	Full cov	yes

Figure 5. Annotation with two concepts with related terms that overlap in one token.

In Figure 6 more than one code is used for the annotation of a single token. "CHOP" is a drug combination that corresponds to: **C**yclophosphamide; **H**ydroxydaunorubicin (doxorubicin); **O**ncovin (vincristine); and **P**rednisone. As there is no single code for "CHOP", the meaning has to be

assembled by a code for each substance. Together, they fully cover the chunk, therefore concept coverage is full. It is self-evident that term coverage is negative.

TOKENS	CHUNK	CODE<br>SNOMED ID	CONCEPT COVERAGE	TERM COVERAGE
CHOP	1	387420009; 372817009; 387126006; 116602009	Full cov	no

Figure 6. Annotation group with four codes assigned to one token.

The Figure shows an example of an empty annotation. Here, the terminology does not have provide a code for the "anginal equivalent", and no code for single tokens "anginal" and "equivalent". Therefore, the code column is empty, and the concept coverage is set "out of scope". If any of both are empty, out of scope is assumed.

TOKENS	CHUNK	CODE<br>SNOMED ID	CONCEPT COVERAGE	TERM COVERAGE
Anginal	1		No cov	no
equivalent	1		No cov	no

Figure 7. Example of empty annotation group

The Figure 8 shows an example of how the term in a terminology does not fully cover the content of a chunk. The term is |28189700|Helicobacter eradication therapy, and the text in the chunk is "eradication of HP". In this case HP is not represented in any entry term of the code in the terminology so it does not cover the text but only the token "eradication" is covered.

TOKENS	CHUNK	CODE<br>SNOMED ID	CONCEPT COVERAGE	TERM COVERAGE
eradication	1	281897000	Full cov	yes
of	1	281897000	Full cov	no
HP	1	281897000	Full cov	no

Figure 8. Example of positive and negative term coverage using one term. The term is |28189700|Helicobacter eradication therapy.

Figure 9 is an example of how to use the inferred coverage score. In the annotation table the chunk number 1 contains the concept chest pain and the chunk number 3 means that the patient's chest pain was treated with nitroglycerin but the patient did not feel any relief. In this case we have two option, we could annotate the token "relief" with the concept "|224978009| relief", or infer that the meaning of the token is "pain relief" because it is related to the chest pain in the chunk number 1.

Besides, the term coverage of the inferred concept is negative due to lack of matching tokens. Here, only the token "relief" matches but the token "pain" is inferred, therefore, the term coverage is negative. Finally, the token "with" in the chunk number 3 is not annotated because this type of tokens is out of scope of the annotations. However, if the token was part of the concept it would be annotated.

TOKENS	CHUNK	CODE SNOMED ID	CONCEPT COVERAGE SCORE	TERM COVERAGE Y/N
Patient				
with				
chest	1	29857009	Full cov	yes
pain	1	29857009	Full cov	yes
and				
MI	2	22298006	Full cov	yes
in				
the				
past				
.				
No				
relief	3	182970005	Inf cov	no
with	3			
nitroglycerin	3	387404004	Full cov	yes

Figure 9. Example of inferred coverage score with context information.

Figure 10 shows the situation when an acronym is not annotated because it is not recognised or its meaning it is not understood. If the context of the acronym does not clarify its meaning the token should not be annotated. Therefore, the chunk, code, concept and term coverage cells related to the token have to remain empty. In case, the AVERBIS term browser retrieves only one concept which has an entry term with the token or acronym, annotators have to be sure that the meaning of the concept and the token matches. Annotators could use Wikis or medical acronym browsers to search for the meaning of the tokens but they cannot use other terminology browsers, only AVERBIS term browser is allowed.

TOKENS	CHUNK	CODE SNOMED ID	CONCEPT COVERAGE SCORE	TERM COVERAGE Y/N
LAD				

Figure 10. Example of fail to annotate a chunk due to misunderstanding of the text.

Annex A

Terminology settings assigned to each language

LANGUAGE	SCT ONLY	UMLS EXT	LOCAL
FRENCH	X	X	
ENGLISH	X	X	
DUTCH	X	X	
SWEDISH	X	X	
FINNISH		X	
GERMAN		X	X
